# Fault Diagnosis Method for Mechanical Components Fusing RPM, DAM-IResNet, and Transfer Learning

**DOI:** 10.3390/s26072162

**Published:** 2026-03-31

**Authors:** Xingwei Ge, Ziyang Chen, Yachao Cao, Zhe Wu, Qi Li

**Affiliations:** School of Mechanical Engineering, Hebei University of Science and Technology, Shijiazhuang 050018, China

**Keywords:** rolling bearings, gear, fault diagnosis, Relative Position Matrix, downsampling attention module, residual network, transfer learning

## Abstract

This paper proposes a novel fault diagnosis method that integrates a Relative Position Matrix (RPM), a Downsampling Attention Module (DAM), an Improved Residual Network (IResNet), and transfer learning to address the challenges of scarce fault data and poor generalization under variable working conditions. The RPM converts 1D vibration signals into 2D images to enhance feature representation. The DAM achieves lossless feature compression and selection via Haar wavelet downsampling and convolutional attention. An IResNet then performs deep feature learning and classification. A transfer learning strategy further enables effective knowledge adaptation from data-rich source domains to data-scarce target domains, significantly improving performance in cross-condition and small-sample scenarios. Experiments on multiple bearing and gear datasets demonstrate that the proposed method achieves over 99.5% accuracy, with 100% in key transfer tasks, outperforming existing state-of-the-art approaches. The main contributions of this work include the unified RPM-DAM-IResNet framework, a targeted small-sample transfer strategy, and comprehensive validation of its superior accuracy and robustness.

## 1. Introduction

With the rapid development of industrial intelligence and automation, rolling bearings, and gearboxes as key components in rotating mechanical systems, their operating statuses directly affect the reliability, safety, and service life of the entire system. During long-term service, all may fail under complex working conditions [1,2]. If not detected and diagnosed in a timely manner, failures lead to unplanned downtime, soaring maintenance costs, and even safety accidents [3]. Therefore, developing high-precision and strong generalization intelligent fault diagnosis methods is of great engineering significance for realizing predictive maintenance of mechanical equipment and ensuring the safe and efficient operation of industrial systems [4]. “Made in China 2025” points out that the global manufacturing industry is facing major adjustments, and China’s manufacturing industry must seize opportunities and actively respond to internal and external challenges. Traditional fault diagnosis methods, which primarily rely on expert experience and basic signal processing, have played a significant role in maintenance for decades. However, under increasingly complex operational conditions, these approaches often struggle to achieve sufficient accuracy in fault feature extraction and diagnosis. Since the 1970s, vibration analysis and machine learning have been increasingly applied to gearbox fault diagnosis, elevating automatic feature learning and decision-making capabilities [5,6,7].

LIU et al. [8] addressed the requirement of a large amount of labeled data for conventional deep learning by leveraging the characteristics of semi-supervised generative adversarial networks. They used unlabeled real data to improve the training effect of labeled data and selected sparse matrices for feature selection, thereby enhancing the classification performance. YANG et al. [9] proposed an oversampling method based on generative adversarial networks for small or imbalanced datasets. Wavelet packet transform was used to generate and extract features, and the data generated by the generative adversarial network was used to supplement the dataset. Finally, machine learning algorithms verified that the generated data maintained the same distribution as the real data. YU et al. [10] proposed a hierarchical algorithm based on stacked long short-term memory (LSTM) networks. Utilizing the memory and forgetting mechanism of LSTM networks, the inherent features of original time signals were automatically extracted through hierarchical stacking of LSTM networks without any preprocessing or manual feature extraction, constructing an end-to-end fault diagnosis system framework. ZHANG et al. [11] combined the advantages of deep belief networks and variational mode decomposition. Variational mode decomposition was used to preprocess original vibration signals to obtain intrinsic mode functions with local information. A deep belief network based on cross-entropy was used to learn the intrinsic mode functions and reconstruct vibration data. Finally, a combination strategy was used to process the data information, achieving more accurate and stable fault diagnosis results. YE Zhuang et al. [12] obtained multi-channel one-dimensional vibration signals by decomposing original vibration signals using empirical mode decomposition. A feature extractor composed of a stacked denoising autoencoder and a multi-channel one-dimensional convolutional neural network was selected to further reduce the dimensionality and extract features of the decomposed one-dimensional signals, realizing feature classification. ZHU et al. [13] proposed a rotor vibration fault diagnosis method based on symmetric dot pattern images and convolutional neural networks. The one-dimensional vibration signal was converted into a two-dimensional image using symmetric dot patterns, and then the convolutional neural network adaptively learned and extracted features from the image to achieve fault classification, effectively diagnosing rotor faults. ZHAO Xiaoqiang et al. [14] designed a multi-scale feature extraction module to maximize the extraction of original signal features and introduced a channel attention mechanism to further extract feature information. Finally, an improved convolutional neural network was used to recognize and classify signals, effectively solving the problems of poor fault diagnosis effect and weak generalization ability of rolling bearings under strong noise environments and variable working conditions. FU et al. [15] tackled the issue where some features in vibration signals are concealed by component noise. Common time-domain parameters were obtained by analyzing vibration signals and combined with an adaptive fuzzy C-means clustering method; some parameters were selected as a new feature vector matrix to obtain more optimized clustering performance. The feasibility and sensitivity of the method were verified through experiments.

However, most existing methods are validated on a single component under specific, stationary conditions. In real industrial settings, mechanical equipment operates under variable loads and speeds, while fault samples for specific modes are scarce. Consequently, existing methods face two intertwined challenges: significant performance degradation due to data distribution shifts across conditions, and insufficient training samples on target tasks to support complex models. Two major challenges persist in current fault diagnosis models. First, under variable working conditions, generalization performance degrades significantly due to shifts in data distribution. Second, on target working conditions or components, the available fault samples are often severely insufficient, which hinders the adequate training of complex models. Although transfer learning has been introduced to mitigate data scarcity, effectively extracting and transferring fault features with strong generalization capability—particularly in cross-component and cross-condition scenarios—remain critical and unresolved difficulties. Although transfer learning has been introduced to alleviate the data scarcity problem, effectively extracting and transferring fault features with strong generalization ability in cross-component and cross-working-condition transfers remains an urgent difficulty to overcome. To address the above issues, this paper proposes a fault diagnosis method fusing Relative Position Matrix, Downsampling Attention Residual Network, and transfer learning. The core idea of the method is as follows: first, the Relative Position Matrix is used to encode one-dimensional vibration signals into two-dimensional images to enhance the expression ability of time series features; second, a feature extraction module combining Haar wavelet downsampling and convolutional attention is designed to achieve lossless screening of multi-scale key features; then, an Improved Residual Network is constructed for deep feature learning and classification; finally, a transfer learning mechanism is introduced to realize knowledge transfer from the source domain with sufficient data to the target domain with scarce data, thereby reducing the dependence on target domain samples and improving the diagnostic robustness of the model under variable working conditions. Compared to existing studies, the proposed method offers distinct advantages: (a) The RPM encoding method preserves the relative temporal relationships within vibration signals more comprehensively than other time–frequency image conversion techniques (e.g., Symmetrized Dot Pattern), providing a richer feature basis for deep networks. (b) The DAM module uniquely combines lossless downsampling via Haar wavelet with attention mechanisms, ensuring that critical high-frequency fault signatures are retained and highlighted during feature compression, unlike conventional pooling or attention modules applied directly to raw features. (c) The tailored transfer learning workflow is specifically designed for the scenario of scarce target domain samples, enabling rapid and stable model adaptation with minimal new data.

In this paper, bearings and gearboxes are taken as typical objects for method verification, aiming to provide a universal and efficient solution for cross-component and cross-working-condition intelligent fault diagnosis of rotating machinery.

## 2. Basic Theory

### 2.1. Relative Position Matrix

**Figure 1:**
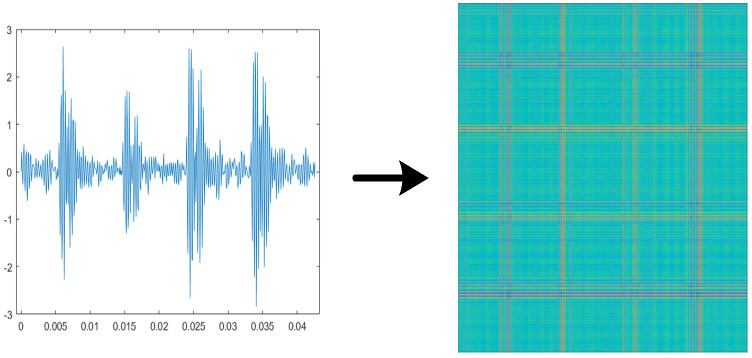
Image of a one-dimensional vibration signal converted by RPM.

The Relative Position Matrix (RPM) [16] is a method for converting time series data into two-dimensional images. Figure 1 shows the two-dimensional image of a one-dimensional vibration signal converted by RPM. When applied to vibration signals, RPM encodes the relative amplitude differences between time points, which correspond to impulsive impacts (e.g., gear tooth breakage) and periodic modulations (e.g., bearing wear). Unlike spectrograms that represent frequency content, RPM retains the temporal structure and enhances the visibility of transient events and cyclic patterns, making it particularly suitable for mechanical fault diagnosis.

First, a time series is represented as $T=t_{1},t_{2},\ldots ,t_{n}$, where *t_i_* is the value at timestamp i, and the length of the time series is n. For the original time series data *T*, z-score standardization is performed to obtain the standard normal distribution **z**:

(1) $$z_{t}=\frac{x_{t}-\mu }{\sigma },\;t=1,2,\ldots ,n,$$

where μ is the mean of *T*; σ is the standard deviation of *T*.

Then, the piecewise aggregate approximation method is adopted to reduce the dimensionality of the normalized time series data from n to m while maintaining the approximate trend of the original sequence by calculating the average value of piecewise constants. A suitable reduction factor k is selected to generate a new smooth time series X ($X=x_{1},x_{2},\ldots ,x_{m}$):

(2) $${\tilde{x}}_{i}=\left\{\begin{array}{c} \frac{1}{k}\sum_{j=k\times \left(i+1\right)}^{k\times i}z_{j},i=1,2,\ldots ,m,\frac{N}{k}-\frac{N}{k}=0\\ \left\{\begin{array}{c} \frac{1}{k}\sum_{j=k\times \left(i+1\right)}^{k\times i}z_{j},i=1,2,\ldots ,m-1\\ \frac{1}{N-k\times \left(m-1\right)}\sum_{j=k\times \left(m-1\right)}^{N}z_{j},i=m \end{array},\frac{N}{k}-\frac{N}{k}>0\right. \end{array}\right.$$

(3) $$m=\frac{N}{k}$$

To handle cases where the length *n* is not perfectly divisible by the reduction factor *k*, we employ a non-overlapping sliding window approach for the piecewise aggregate approximation, truncating the remainder of the sequence. The resulting grayscale matrix M of size m × m is then resized to a standard dimension of 224 × 224 pixels using bilinear interpolation to serve as the network input. Subsequently, the preprocessed time series *X* is converted into a two-dimensional matrix *M* by calculating the relative position between two timestamps:

(4) $$M=\left[\begin{array}{cccc} {\tilde{x}}_{1}-{\tilde{x}}_{1} & {\tilde{x}}_{2}-{\tilde{x}}_{1} & \ldots & {\tilde{x}}_{m}-{\tilde{x}}_{1}\\ {\tilde{x}}_{1}-{\tilde{x}}_{2} & {\tilde{x}}_{2}-{\tilde{x}}_{2} & \ldots & {\tilde{x}}_{m}-{\tilde{x}}_{2}\\ \vdots & \vdots & \ddots & \vdots \\ {\tilde{x}}_{1}-{\tilde{x}}_{m} & {\tilde{x}}_{2}-{\tilde{x}}_{m} & \ldots & {\tilde{x}}_{m}-{\tilde{x}}_{m} \end{array}\right]$$

Obviously, every two timestamps of the time series are connected to obtain their relative positions. Each row and column of M contains information of the entire time series with a certain timestamp as the reference point. In addition, as an advantage of this method, RPM can be regarded as a data augmentation method to improve generalization ability by providing redundant features of the time series. Each row of M shows the time series with different reference points, and each column shows the mirror image of the former, thereby providing a reverse perspective to observe the time series. Finally, min-max normalization is used to convert M into a grayscale matrix, and the Relative Position Matrix is obtained, as follows:

(5) $$F=\frac{M-min\left(M\right)}{\max\left(M\right)-min\left(M\right)}\times 255$$

The image converted by this method contains redundant features of the original time series, making it easier to capture the similarity information between and within classes, which can be better extracted for features and achieve better performance. Although existing 2D encoding methods (e.g., Gramian Angular Field, Markov Transition Field) have been applied to fault diagnosis, they exhibit certain limitations: GAF relies on polar coordinates and may distort high-frequency components, while MTF depends on quantile partitioning and can lose fine-grained temporal details. In contrast, the Relative Position Matrix (RPM) preserves the relative temporal order and amplitude differences directly, making it more suitable for representing periodic and impulsive features in vibration signals.

### 2.2. Downsampling Attention Module

After the data is input into the convolutional neural network, to reduce computational cost, increase the receptive field, and promote feature extraction, the data first passes through the initial downsampling module to reduce its size to 1/4 of the original, which helps the network better learn complex features and achieve better performance. However, such initial downsampling will reduce the spatial information and spatial resolution of the input data, affecting the network’s ability to represent the input data. Therefore, a new initial downsampling method is needed to solve the above problems. The Downsampling Attention Module (DAM) is mainly used to achieve lossless scaling of input features and screen features according to their importance. It consists of a Convolutional Attention Module (CAM) [17] and Haar wavelet downsample (HWD) [18].

**Figure 2:**
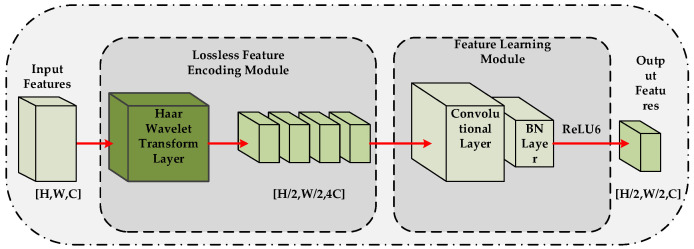
Structure diagram of the HWD module. Arrows indicate the direction of feature map flow during the downsampling process.

As shown in Figure 2, the HWD module first decomposes the input feature map into low-frequency approximation and high-frequency detail components using Haar wavelet transform. This transform is a mathematically invertible lossless operation: while halving the feature size, it fully preserves all information in the original features (including high-texture details). Then, a lightweight convolutional layer is used to fuse and adjust the decomposed multi-channel features.

**Figure 3:**
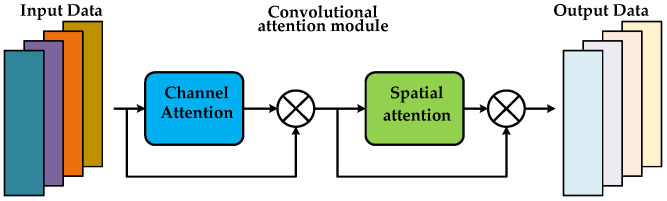
Overall structure of the Convolutional Attention Module.

As shown in Figure 3, for the vibration signal feature maps (derived from HWD’s lossless processing) in this fault diagnosis task, the Convolutional Attention Module adaptively weights the importance of different channels and spatial positions via dual-channel–spatial attention: it amplifies the weights of subtle fault-related features (e.g., vibration textures corresponding to bearing outer ring wear or gear tooth breakage) while suppressing irrelevant background noise, allowing the network to precisely focus on fault-correlated regions. This enables targeted intelligent feature screening and enhancement based on the lossless feature base provided by HWD.

The DAM module processes features in a carefully designed sequence. The HWD component first performs lossless downsampling via invertible wavelet transform, preserving crucial high-frequency details (e.g., impact transients) that are often filtered out by standard pooling. This yields a compact yet information-complete feature base. Subsequently, the CAM component applies attention mechanisms to this refined base, selectively amplifying the most discriminative fault-related features. This sequence ensures that attention is focused on a meaningful, information-preserved representation, rather than being applied to potentially noisy or redundant raw features, thereby enhancing both efficiency and feature selection quality. The innovation of the DAM module lies in the sequential combination of HWD and CAM. It first provides the network with a feature base with complete information, and then performs precise feature screening on this basis, thereby significantly enhancing the model’s ability to extract fault features while improving computational efficiency.

### 2.3. Improved Residual Module

The Residual Network (ResNet) addresses degradation in deep networks, enabling the construction of sufficiently deep models—a critical capability for learning complex, hierarchical fault features (such as subtle wear textures and crack-induced vibration components) from non-stationary mechanical vibration signals. In real industrial monitoring scenarios, such as in-process geometric feature classification for machining quality control, deep networks often suffer from performance saturation or degradation as depth increases. The residual structure effectively mitigates this issue by preserving shallow features through identity mapping, making it particularly suitable for tasks involving gradual feature evolution, such as wear progression or crack propagation in mechanical components. To mitigate degradation issues like gradient vanishing and overfitting caused by increasing network depth, He Kaiming et al. proposed the Residual Block (RB) [19], which consists of convolutional layers, BN layers, and ReLU activation functions. Convolutional layers extract fault-related features from vibration signal feature maps; BN layers normalize the feature map data of vibration signals, alleviate gradient dispersion, and enhance model stability; the ReLU activation function strengthens the model’s ability to fit nonlinear relationships in fault features. The synergy of these structures allows the residual module to converge quickly when processing non-stationary vibration signals, while improving the module’s generalization performance and diagnostic accuracy.

**Figure 4:**
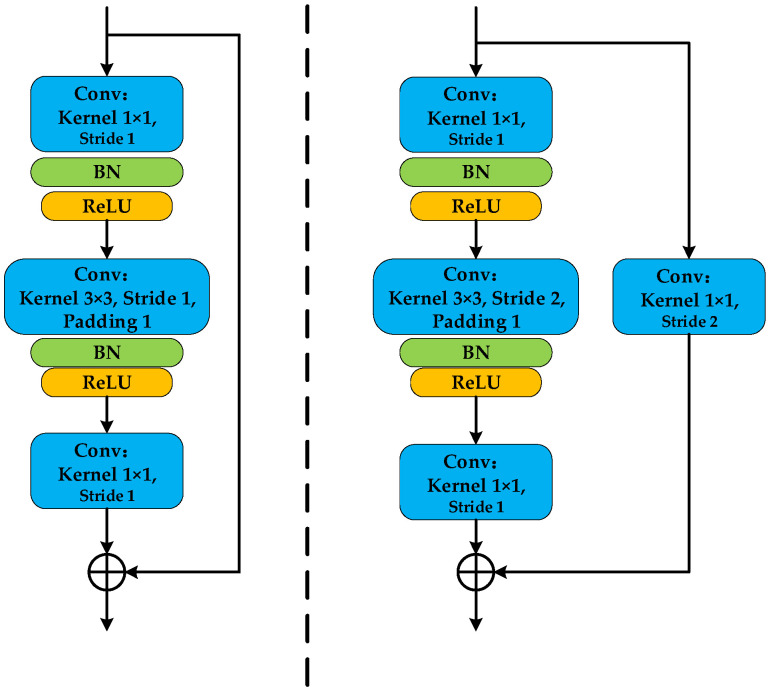
Structure of the traditional residual module.

The identity mapping branch superimposes the initial input of the residual module (which contains shallow, weak fault features of vibration signals) with the output after multi-layer convolution. This avoids the loss of shallow information caused by deeper networks without introducing additional parameters, effectively resolving the degradation problem of deep neural networks in fault feature extraction tasks. The structure of the traditional residual module is shown in Figure 4.

**Figure 5:**
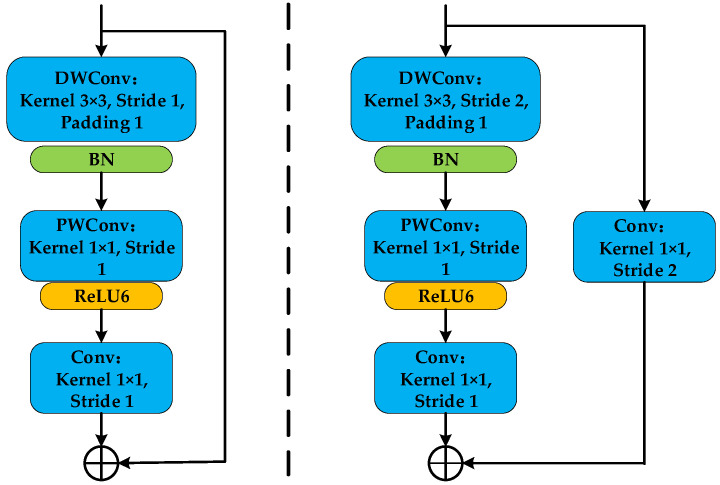
Improved Residual Module schematic.

This paper proposes an Improved Residual Block (IRB) to enhance feature extraction ability while maintaining efficiency. The design of the IRB draws inspiration from the inverted residual block of MobileNetV2 [20], which employs a depthwise separable convolution followed by pointwise expansion and projection. As shown in Figure 5, our IRB is mainly composed of convolutional layers, BN layers, and ReLU6 activation functions. ReLU6 is a variant of the ReLU activation function, and its expression is:

(6) $$\mathrm{ReLU}6\left(x\right)=\min\left(\max\left(0,x\right),6\right)$$

This function can effectively prevent gradient explosion and is commonly used in lightweight architectures.

The detailed modifications in our IRB are as follows: First, the standard convolution is replaced with a more parameter-efficient depthwise separable convolution. A 3 × 3 depthwise convolution serves as the first layer, followed by a 1 × 1 pointwise (PW) convolution layer for channel expansion. A BN layer is inserted between these two convolutional layers. The expanding pointwise convolution increases the channel number (e.g., to 4 times the input), after which a ReLU activation function introduces non-linearity. Finally, another 1 × 1 convolution reduces the channels back to match the input dimension. The identity mapping branch adds the initial input to the processed features, preserving the residual learning framework.

Compared to the traditional residual module, the key distinctions and contributions of our IRB in the context of this work are: (1) it integrates the lightweight inverted residual design principle into a stable residual learning framework suitable for deep feature extraction from vibration images; (2) it employs ReLU6 activation to enhance training stability within this adapted structure; and (3) it serves as the core building block of our custom DAM-IResNet backbone, enabling the construction of a model that is both accurate and computationally efficient for fault diagnosis tasks.

### 2.4. Transfer Learning

Gearbox fault diagnosis requires a large amount of data to train the model, but fault data is difficult to obtain in practice. To solve this problem, a method combining the Downsampling Attention Residual Network and transfer learning [21] is proposed. Transfer learning applies the knowledge obtained in one task to another task, improving the model’s performance in the new field and reducing the demand for data in the new field.

To address the insufficient model training caused by scarce fault samples in the target domain, this paper adopts model-based transfer learning. Its core lies in transferring the knowledge learned from the source domain to the target domain through three steps: pre-training, transfer, and fine-tuning. The specific steps are as follows:

(1) Source domain pre-training

The proposed DAM-IResNet network is trained on the source domain dataset $D_{s}=\{(x_{s}^{\left(i\right)},y_{s}^{\left(i\right)}){\}}_{i=1}^{N_{s}}$. The training goal is to minimize the classification loss (cross-entropy loss) of the source domain:

(7) $$L_{s}=-\frac{1}{N_{s}}\sum_{i=1}^{N_{s}}\sum_{c=1}^{C_{s}}1\left[y_{s}^{\left(i\right)}=c\right]\log p_{s}^{\left(i\right)}\left(c\right)$$

where $C_{s}$ is the number of fault categories in the source domain, and $p_{s}^{\left(i\right)}(c)$ is the prediction probability that the model predicts the sample $x_{s}^{\left(i\right)}$ belongs to category c. This stage enables the model to learn general fault feature representations.

(2) Model transfer

The parameters $\theta_{\mathrm{feat}}$ of all feature extraction layers are retained in the pre-trained model (including RPM encoding, DAM module, and IRB backbone network) and its classification layer is removed. $\theta_{\mathrm{feat}}$ is used for the initial feature extraction parameters of the target domain model.

(3) Target domain fine-tuning

Two-stage fine-tuning is performed on the target domain dataset $D_{t}=\{(x_{t}^{\left(j\right)},y_{t}^{\left(j\right)}){\}}_{j=1}^{N_{t}}$ (usually $N_{t}\ll N_{s}$):

Freeze features: The parameters $\theta_{\mathrm{feat}}$ are frozen, and the randomly initialized new classification layer is trained only on parameters $\theta_{\mathrm{cls}}^{*}$.

Joint fine-tuning: Both $\theta_{\mathrm{feat}}$ and $\theta_{\mathrm{cls}}^{*}$ are fine-tuned, with a small learning rate to minimize the target domain cross-entropy loss:

(8) $${{min}_{\theta_{feat},\;\theta_{cls}}L}_{s}=-\frac{1}{N_{t}}\sum_{j=1}^{N_{t}}\sum_{k=1}^{C_{t}}1\left[y_{t}^{\left(j\right)}=k\right]\log p_{t}^{\left(j\right)}\left(k\right)$$

The algorithm realizes the effective transfer of feature knowledge, enabling the model to achieve excellent diagnostic performance even when the amount of target domain data is limited.

## 3. Construction of Fault Diagnosis Model

### 3.1. Model Structure and Parameters of Downsampling Attention–Residual Network

**Figure 6:**
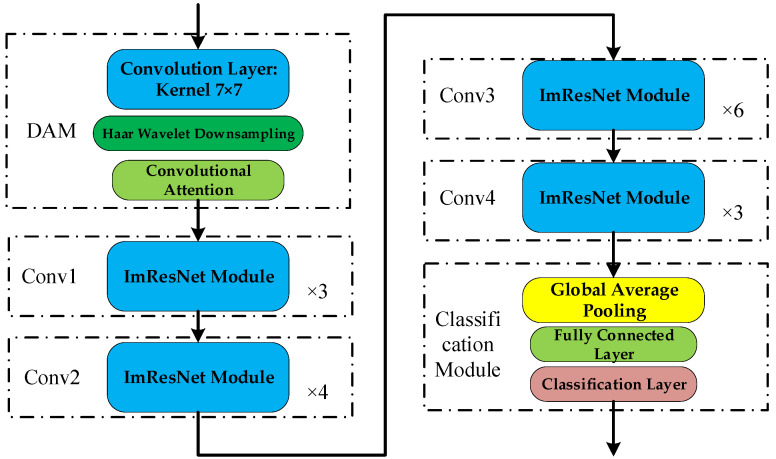
Overall architecture of the DAM-IResNet model.

The overall architecture of the Downsampling Attention–Residual Network (DAM-IResNet) is illustrated in Figure 6. It comprises three core components: the Haar Wavelet Attention Module (HWAM) for initial feature extraction and compression, the backbone network (Stages Conv1–Conv4) constructed by stacking Improved Residual Blocks (IRBs) for deep feature learning, and the classification module for prediction.

The precise configuration of each stage—including input dimensions, the operation sequence within each building block, the number of repetitions, and the output dimensions—is summarized in Table 1. In the table, DSC denotes a depthwise separable convolution, and → indicates the sequential flow of operations within an IRB.

To quantitatively evaluate the efficiency improvement achieved by the proposed DAM module and the overall network design, we compare the computational cost of DAM-IResNet with the baseline ResNet50 and a representative Transformer-based model, Vision-Transformer, under identical conditions. The results are summarized in Table 2. The comparisons in terms of parameter count, FLOPs, and inference time per sample all demonstrate that, thanks to the lightweight attention module and the improved residual structure design, DAM-IResNet significantly reduces model complexity and computational burden while maintaining high performance. It shows particularly strong potential for practical deployment compared to the Transformer architecture.

### 3.2. Fault Diagnosis Flow Based on RPM and DAM-IResNet

**Figure 7:**
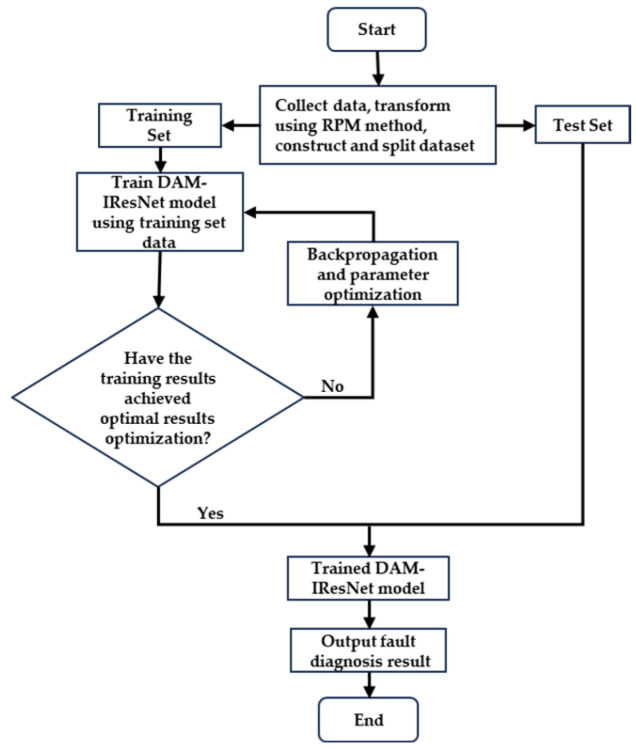
RPM-DAM-IResNet fault diagnosis flowchart.

This paper proposes a gearbox fault diagnosis method based on RPM and Improved Residual Network. The flow chart of the method is shown in Figure 7. First, the vibration signals of the gearbox are collected; then, the one-dimensional vibration signals are converted into two-dimensional images according to a certain data length using the RPM method, constructing a fault classification dataset, which is divided into a training set and a validation set; the training set images are input into DAM-IResNet for training, and the hyperparameters in the network are adjusted according to the training results until the results converge; finally, the test set is input into the network model with converged training results for verification to obtain the final fault classification results.

### 3.3. Fault Diagnosis Flow Integrating Transfer Learning

**Figure 8:**
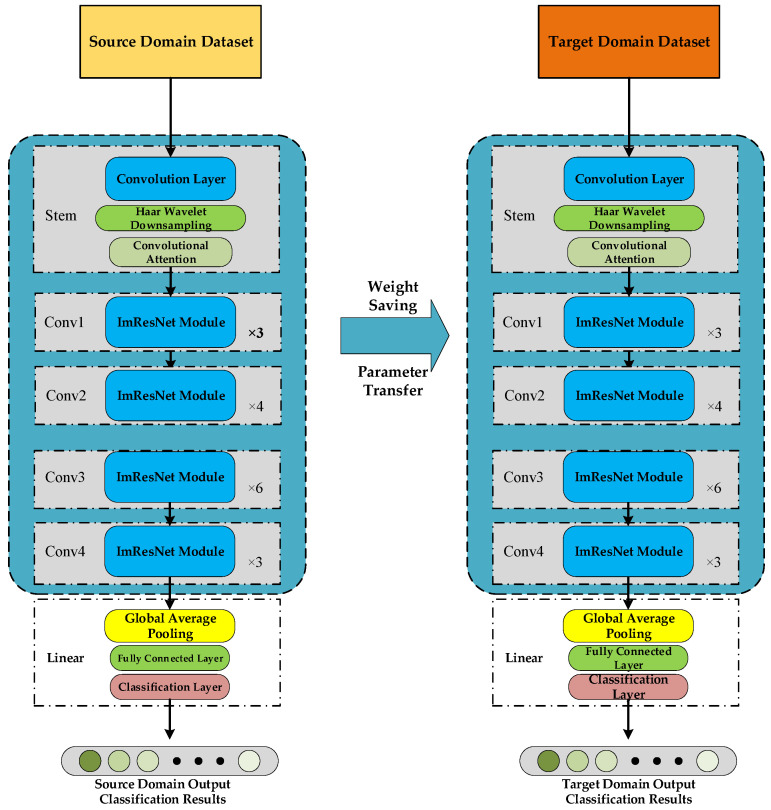
RPM-DAM-IResNet transfer learning fault diagnosis flow chart.

The fault diagnosis model based on DAM-IResNet can reduce the dimensionality of input data and extract feature representations highly relevant to classification tasks through the Downsampling Attention Module combined with HWD and CBAM. Then, the ImResNet network further screens the extracted features to remove irrelevant information. Considering the demand for cross-working-condition fault diagnosis, combined with the idea of transfer learning, the model weights fully trained on the source domain dataset are transferred to the target domain dataset to complete the model of the target transfer task. In summary, a gearbox fault model based on RPM-DAM-ImResNet and transfer learning is proposed, and the specific structure of the model is shown in Figure 8. The flow chart of the method used in the model is shown in Figure 8. First, vibration signal data is collected; then, the RPM method is used to realize image encoding of the data to obtain two-dimensional images, which are divided into source domain dataset and target domain dataset according to requirements. The two datasets are respectively divided into training set and validation set. The DAM-IResNet network is fully trained using the source domain data training set. After the model training meets the expectations, the transferable pre-trained network structure is retained using the model transfer method, and the classification layer is reconstructed according to the target task. The reconstructed network model after transfer is fully trained using the target domain data training set until the training meets the expectations, and the network is verified using the target domain data validation set to complete the fault diagnosis task.

## 4. Experimental Verification

### 4.1. Experimental Data

**Figure 9:**
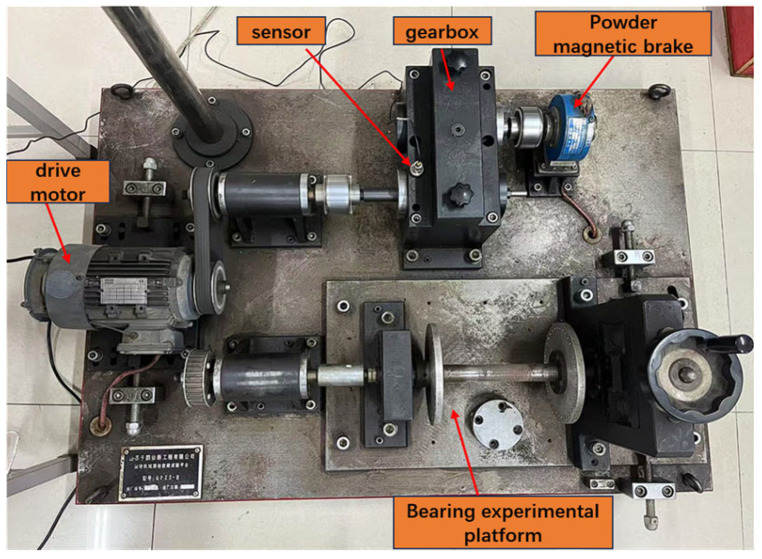
QPZZ-II test bench.

To fully verify the effectiveness of the proposed method, a dataset was constructed using the gearbox vibration signal data collected from actual experiments for experimental research. The experimental platform used in the experiment was the QPZZ-II test bench, as shown in Figure 9. This test bench can quickly simulate various states and vibrations of rotating machinery, as well as perform state analysis and fault diagnosis. The test bench is composed of a variable-speed drive motor, bearings, a gearbox, a governor, etc. Various faults can be quickly simulated by adjusting the counterweight, the installation position of the adjustment part, and the organic combination of components.

The gears of the gearbox on the test bench are spur gears. The pinion on the input shaft has 55 teeth (Z1 = 55), and the number of teeth of the gearwheel on the output shaft Z2 = 75. The sampling frequency Fs = 5200 Hz. The experiment includes 4 channels of collected signals, namely the motor side and load side of the input shaft, and the motor side and load side of the output shaft. The gear working conditions were healthy gear, gear tooth breakage, gear wear, root crack, and surface pitting. Two working conditions were set for the experiment through load: no-load and fixed load of 5 Nm. An experimental dataset was constructed based on the collected data, named Dataset D, which was designated as D0 and D5 according to different working conditions. The specific data is shown in Table 3.

**Figure 10:**
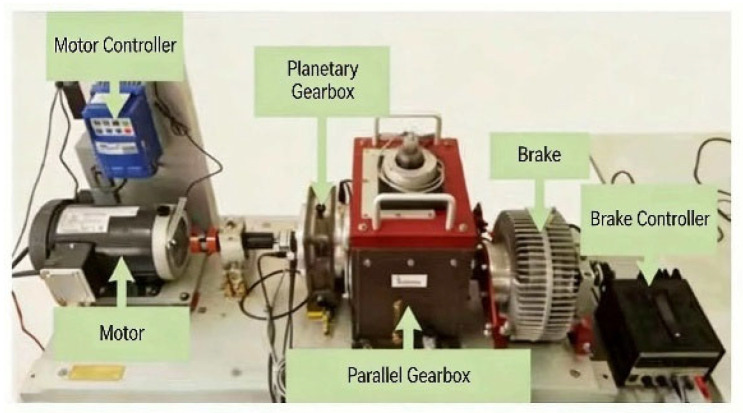
Dynamic simulator of the transmission system of Southeast University.

The gearbox dataset from Southeast University (SEU) [22] was selected to verify the proposed network model. The data of the SEU dataset was derived from the Drivetrain Dynamic Simulator (DDS), whose structure is shown in Figure 10. The DDS has two gearboxes, and the experimental data was from the planetary gearbox among them. A total of 7 vibration sensors were used on the test bench to collect data, among which 3 sensors received the vibration signals of the planetary gearbox in the x, y, and z directions respectively.

The SEU dataset includes a bearing dataset and a gear dataset. The bearing dataset includes five states of bearings: healthy, rolling element fault, inner ring wear, outer ring wear, and combined fault of inner and outer rings; the gear dataset includes five states of gears: healthy, gear tooth breakage, missing gear tooth, root crack, and surface pitting. The gear faults under two different working conditions were studied when the load of the speed system was set to 20HZ-0V or 30HZ-2V. To make full use of the SEU dataset, an experimental dataset was constructed. The construction rules are as follows: the gear dataset was used to construct Class A datasets, named A0 and A2 according to different working conditions; the bearing dataset was used to construct Class B datasets, named B0 and B2 according to different working conditions. The specific content of the SEU dataset is shown in Table 4.

**Figure 11:**
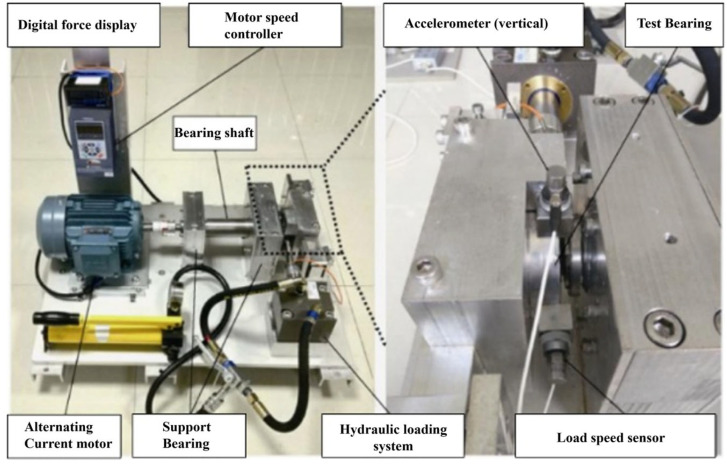
Rolling Bearing Data Acquisition Laboratory, Xi’an Jiaotong University (XJTU).

The test bench structure of the XJTU full-life cycle rolling bearing dataset from Xi’an Jiaotong University is shown in Figure 11. A hydraulic loading system was used to generate radial force, and the speed was adjusted through the speed controller of the AC motor. LDK UER204 rolling bearings were selected as the experimental objects. Two PCB-352C33 unidirectional acceleration sensors were used to collect the vibration signals of the test bearings in the vertical and horizontal directions respectively, and the sampling frequency was set to 25.6 kHz. XJTU-SY is full-life data. To construct the classification dataset shown in Table 4, samples were extracted according to the following principles: healthy state samples were taken from the data segments of the bearing at the initial stage of operation, with good performance and no fault signs; samples of each fault state (such as outer ring and inner ring faults) were taken from the data segments where the fault clearly occurred and entered a stable development period in the accelerated life test, to avoid unstable data at the time of fault initiation or complete failure.

According to the speed and radial force, the dataset was divided into 3 types of working conditions. Based on the 3 types of working conditions and the obtained bearing information data, the used bearing dataset C was constructed. Considering that the dataset contains the full-life cycle data of bearings, the data of the outer ring fault under the 3 types of working conditions when the bearing had not yet failed were selected as healthy data and added to the dataset. The specific division of Class C dataset is shown in Table 5.

**Figure 12:**
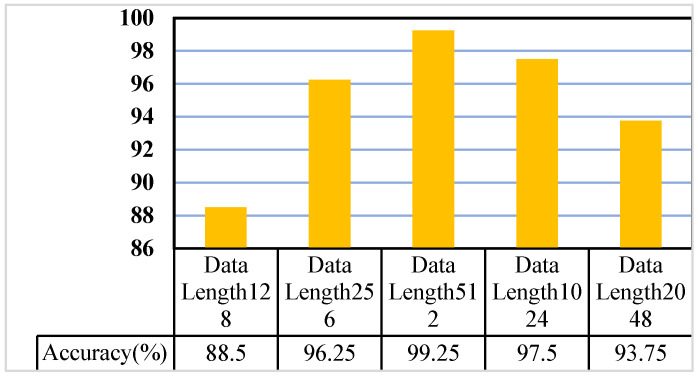
Effect of data encoding length on classification accuracy.

Taking the A0 gear dataset as an example, the RPM method was used for data preprocessing. One-dimensional vibration data of different lengths were selected for encoding, and experiments were carried out using the model used in this section. The experimental results are shown in Figure 12. It can be seen from Figure 12 that, when the data length is 512, the test model has the highest accuracy.

To ensure the consistency and reproducibility of the experiments, a unified data construction and splitting protocol was applied to all datasets (SEU, XJTU, QPZZ-II). The protocol was designed to preserve the independence between training and validation samples through two key steps. First, during sample construction, non-overlapping sliding windows were applied to the raw vibration signals. Crucially, the source data for each fault condition comprised multiple, physically independent experimental runs, meaning samples originated from separate start–stop cycles of the machinery, ensuring inherent diversity. Second, for a balanced and comparable evaluation consistent with common benchmarks, the sample pools from all categories and runs were combined. This aggregated pool was then randomly divided into training and validation sets at an 8:2 ratio (e.g., 800 training and 200 validation samples per fault state in the A0 dataset, as detailed in Table 6). We emphasize that all models evaluated in this study—including the proposed DAM-IResNet and all baseline methods—were trained and tested under this identical data splitting scheme, guaranteeing a fair comparison of architectural performance. This standardized protocol was consistently used across all ablation, variable-condition, and transfer learning experiments.

**Figure 13:**
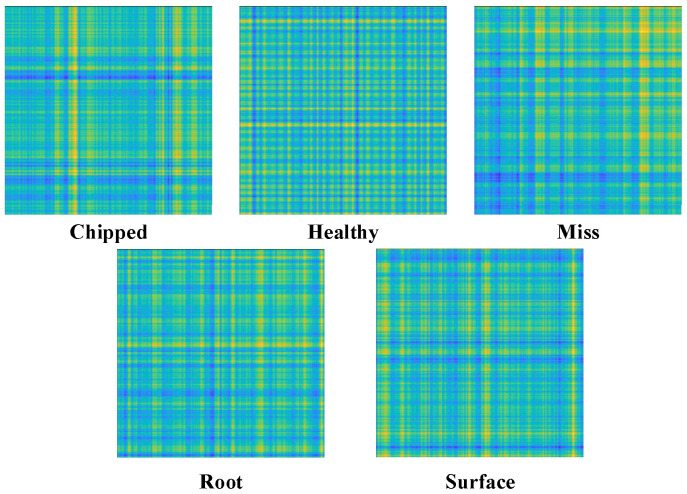
RPM charts corresponding to different faults.

To establish the connection between original physical phenomena and feature encoding, Figure 13 presents the raw vibration time-domain waveforms of five typical states in the A0 dataset and their corresponding RPM encoding results. In the raw waveforms, the healthy state exhibited no obvious impacts and stable amplitude; in contrast, fault states (e.g., chipped teeth, surface pitting) showed characteristic changes—chipped teeth corresponded to periodic impact signals, surface pitting was manifested by increased high-frequency vibration components, and missing teeth resulted in irregular amplitude gaps. By preserving the relative positional relationships of the time series, RPM encoding converted these physical features into grayscale distribution differences in 2D images (e.g., fault regions exhibited significant grayscale contrast in RPM charts), laying a solid foundation for the subsequent network to extract discriminative features.

**Figure 14:**
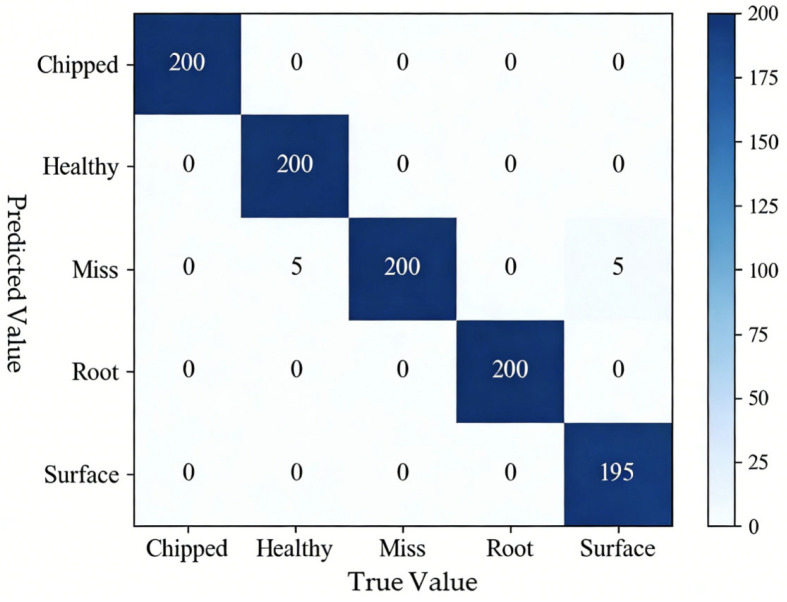
Confusion matrix of the RPM-DAM-IResNet50 method.

Taking the A0 dataset as an example, Figure 14 illustrates that the method combining RPM and DAM-IResNet50 performed excellently on the A0 dataset. It accurately predicted chipped tooth faults, healthy states, and root crack faults, with only one type of misclassification: five surface pitting fault samples were misidentified as missing tooth faults.

In summary, the RPM image encoding method demonstrates outstanding performance in fault diagnosis research. It enables the network to better learn fault features, thereby achieving more effective fault identification and classification.

### 4.2. Fault Diagnosis Performance Evaluation Indicators

To comprehensively evaluate the reliability of the proposed method in gearbox fault diagnosis, this paper used accuracy, precision, recall, and confusion matrix to evaluate the classification performance of the model. The accuracy was automatically given after the network training was completed. The confusion matrix was drawn using the weights generated by the trained network, and the precision and recall were calculated using the data in the confusion matrix. The calculation formula of the evaluation indicator precision is as shown in Equation (9):

(9) $$Precision=\frac{TP}{TP+FP}$$

The calculation formula of the evaluation indicator recall is as shown in Equation (10):

(10) $$Recall=\frac{TP}{TP+FN}$$

where TP is the number of samples where both the true label and the predicted label are positive; TN is the number of samples where both the true label and the predicted label are negative; FP is the number of samples where the true label is negative and the predicted label is positive; FN is the number of samples where the true label is positive and the predicted label is negative; Precision is the proportion of correctly predicted positive samples among all predicted positive samples; Recall is the proportion of correctly predicted positive samples among all positive samples. To ensure a fair comparison of network architectures, rather than preprocessing techniques, all comparative models (ResCNN, MobileNet, Vision-Transformer, etc.) were fed with the same RPM-encoded 2D images as the proposed DAM-IResNet.

### 4.3. Experimental Research and Result Analysis

To verify the effectiveness of the HWD and CAM sub-modules in the DAM module and the IRB module in the proposed DAM-IResNet network model, ablation experiments were used to analyze the three modules. According to the presence or absence of the three modules in the network, the following models were set up as comparative analysis models: Model 1 was the ResNet50 network model; Model 2 was the IResNet50 network model using IRB; Model 3 was CBAM-IResNet, which adds the CBAM module to the IResNet50 network model; Model 4 was HWD-IResNet, which adds the HWD module to the IResNet50 network model; the proposed model was set as Model 5. The model settings are shown in Table 7. Different datasets were selected for the experiments. The optimizer of the experiment was set to Adam, the loss function was set to cross-entropy, the classifier was set to Softmax, the learning rate was set to 0.0001, the batch size was set to 32, and the number of iterations was set to 50. The experiments were carried out on a computer configured with Windows10 operating system, Intel Core i7-9700K processor, and 64 GB memory, using the Pycharm platform, with Python as the programming language, in the Pytorch environment. Under the same working conditions of the training set and the validation set, the QPZZ-II gearbox dataset was used for ablation verification. The accuracy of the ablation experiment under the same working conditions is shown in Table 8.

As shown in Table 8, all models achieved high accuracy under identical working conditions, indicating the effectiveness of RPM encoding in providing a discriminative input representation. Notably, the proposed DAM-IResNet attained the highest accuracy. The progressive improvement from ResNet50 to IResNet50, then to HWD/CBAM variants, and finally to the full DAM-IResNet, validates the contribution of each component: IRB enhances feature learning capacity, HWD preserves critical high-frequency information during downsampling, and CAM enables intelligent feature selection. Their synergy in DAM-IResNet allows for optimal feature extraction and filtering, which is crucial for generalizing to variable conditions.

**Figure 15:**
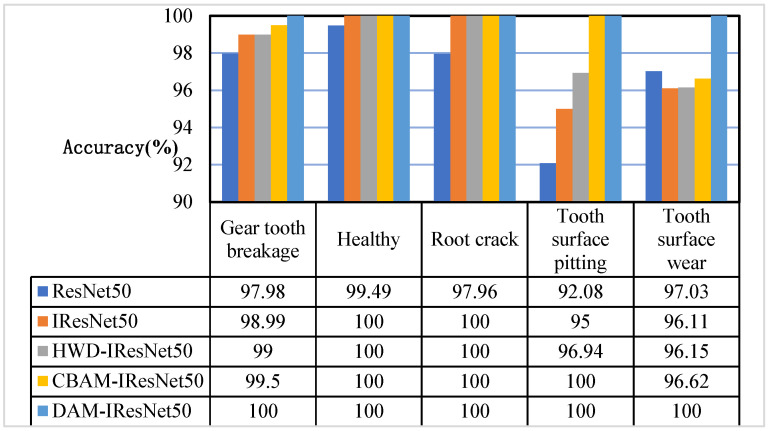
Comparison of the accuracy of the five network models.

To further study the classification and generalization performance of the network model, the precision and recall of the models were analyzed and compared according to the training results of the five network models on the gear dataset C0. The comparison diagrams of the precision and recall of the five network models are shown below. It can be seen from Figure 15 that, in the experiment on the QPZZ-II gearbox dataset, the recognition precision values of the five models for various states were all above 90%. Among them, the ResNet50 network had the lowest recognition precision for surface pitting, which was 92.8%, and there were cases where samples of the other four states were misjudged as surface pitting; the IResNet50 network had the same problem, but, unlike the ResNet50 network, the IResNet50 network could accurately judge the healthy state and root crack state, and the misjudged samples of surface pitting came from the remaining two fault states; adding the HWD module further improved the recognition accuracy of the network for gear tooth breakage, root crack, and surface wear; adding the CBAM module enabled the network to accurately judge the surface pitting fault and further improved the judgment accuracy for gear tooth breakage and surface wear; the proposed method realized accurate recognition of all states.

**Figure 16:**
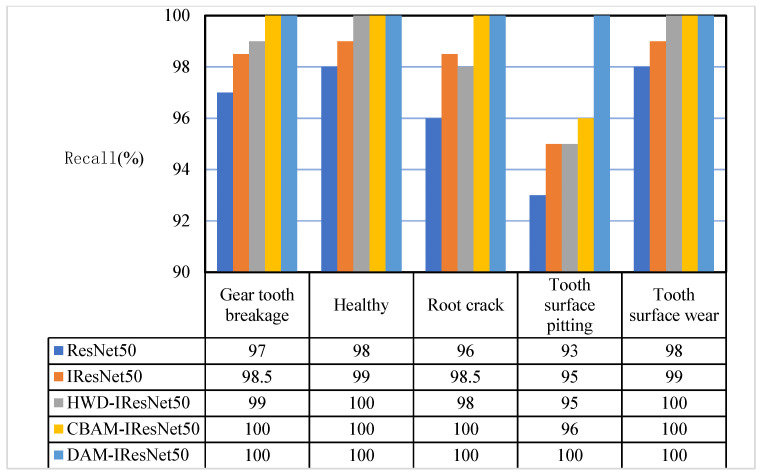
Comparison of recall ratios of the five network models.

It can be seen from Figure 16 that the recall rates of the five models for various states were all above 90%. Among them, the ResNet50 network had the lowest recall rate for surface pitting, which was 93%, and there were cases where samples were misjudged as the other four states; the IResNet50 network had the same problem. Compared with the ResNet50 network, the recall rate of the IResNet50 network for various states was improved, and the recall rate of surface pitting was increased to 95%; adding the HWD module enabled the network to accurately recognize the healthy state; adding the CBAM module enabled the network to accurately recognize all states except the surface pitting fault and further improved the recognition accuracy of surface pitting; the proposed method realized accurate recognition of all states. From the above analysis, it can be seen that the three proposed modules all played an important role in the extraction and classification of gear fault features, and the proposed model could fully learn the fault features of gears and achieve perfect classification of operating states.

### 4.4. Experimental Result Analysis Under Variable Working Conditions

Considering the extremely complex actual working environment of gearboxes, to comprehensively verify the practicality and universality of the proposed method under variable working conditions, some advanced fault diagnosis methods were selected as comparative methods for experimental verification under different working conditions. The comparative methods included ResCNN [23], MobileNet [24], and Vision-Transformer. Among them, ResCNN is a fault diagnosis method based on residual network, MobileNet is a fault diagnosis method based on DSConv, and Vision-Transformer is a fault diagnosis method based on Transformer structure. These methods represent the research directions of deep convolution optimization, lightweight design, and cutting-edge attention mechanisms in the current field of fault diagnosis, respectively.

The verification method was conducted as follows: after training with the source working condition data, the target working condition data was directly tested on, without any fine-tuning to verify the inherent generalization ability of the model features. For the SEU dataset, the following 4 variable working condition experiments were performed, which followed the principle of transfer learning verification for the same type and different working conditions—A0-A2, A2-A0, B0-B2, and B2-B0, where A0-A2 means the A0 dataset was used for model training and the A2 dataset was used for model validation. For the XJTU dataset, the following 2 variable working condition experiments were performed: C0-C1 and C0-C2. For the QPZZ-II dataset, the following 2 variable working condition experiments were performed: D0-D5 and D5-D0. The average accuracy of various models under variable working conditions obtained from the experiments is shown in Table 9.

Table 9 demonstrates that the proposed method consistently outperforms ResCNN, MobileNet, and Vision-Transformer across all variable-condition tasks, and this superiority stems from a physically interpretable feature extraction process that aligns with the intrinsic nature of mechanical faults, rather than mere structural differences. ResCNN adopts standard pooling for downsampling, which inevitably discards high-frequency fault signatures (e.g., transient impacts from gear tooth breakage, cyclic modulations from bearing wear). These are precisely the critical features for distinguishing faults under variable loads and speeds. Its generic convolutional layers lack targeted selection mechanisms, leading to the mixing of fault-related information with spurious noise induced by changes in operational conditions, thus reducing generalization. MobileNet’s depthwise separable convolution, while lightweight for deployment, failed to prioritize fault-relevant features due to the absence of an attention mechanism; it could not effectively distinguish between physically meaningful signals (such as the amplitude differences caused by root cracks) and irrelevant amplitude fluctuations from load variations, resulting in coarse and less discriminative feature representations. Even Vision-Transformer, with its powerful global attention mechanism, overemphasized long-range dependencies in the signal and overlooked localized, fine-grained fault patterns—mechanical faults like surface pitting often manifested as subtle high-frequency textures, which were easily ignored by global attention that focused on overall signal trends.

In contrast, the proposed DAM-IResNet method achieved superior performance by integrating components that work synergistically to capture and emphasize interpretable fault-related features. The Relative Position Matrix (RPM) serves as the foundation by encoding 1D vibration signals into 2D images while preserving relative amplitude differences and temporal order—these encoded features directly map to observable physical phenomena, such as periodic impacts from chipped teeth translating to distinct grayscale contrast in RPM images, making the origin of model-learned features traceable and verifiable. Building on this, the Haar wavelet downsampling (HWD) module in the DAM performs lossless decomposition, retaining all high-frequency detail components that standard pooling filters out; its mathematical invertibility ensures no physically meaningful fault information is lost during dimensionality reduction, providing a complete feature base for subsequent processing. The Convolutional Attention Module (CAM) then adaptively weights channels and spatial regions on this lossless feature base, amplifying the importance of fault-correlated regions (such as the grayscale spikes in RPM images corresponding to gear wear) while suppressing background noise. This attention mechanism’s effectiveness can be visually validated through explainable AI techniques like Grad-CAM, which reveals that the model consistently focuses on physically meaningful fault signatures, rather than random signal fluctuations. Finally, the stacked Improved Residual Blocks (IRBs) deepen the learning of these attended features, leveraging depthwise separable convolution and ReLU6 activation to enhance feature extraction efficiency without sacrificing stability, ensuring the model captures hierarchical fault patterns from subtle wear to severe cracks.

**Figure 17:**
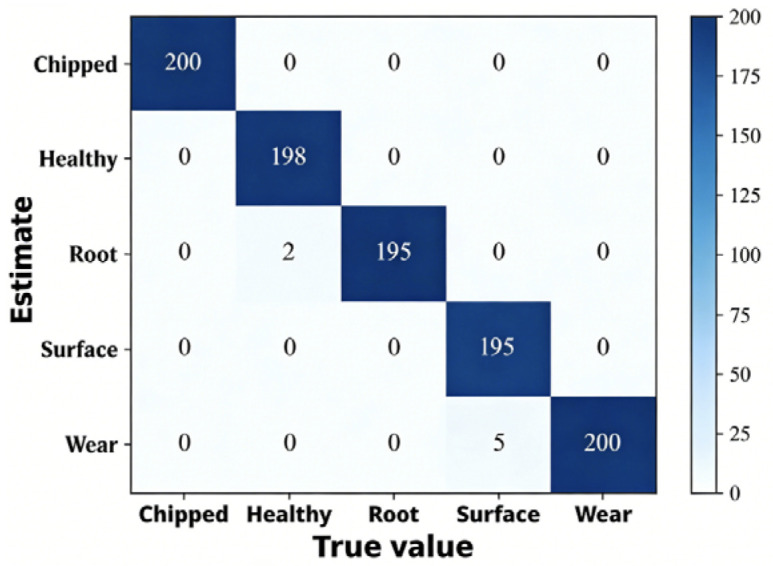
Confusion matrix under D0-D5 variable conditions.

In addition, the confusion matrix of the proposed method under the D0-D5 variable working condition is shown in Figure 17. It can be seen from the Figure that the method combining RPM and DAM-IResNet performs well under the D0-D5 variable working condition and can accurately predict gear tooth breakage faults. There are two types of misjudgments: 2 healthy state samples were judged as root crack faults, and 5 surface pitting samples were judged as missing gear tooth faults. Using the confusion matrix, the RPM- DAM-IResNet method was further analyzed. The precision and recall of the method are shown in Table 10.

The superior performance of our method was further reinforced by the synergistic effect of its components: HWD preserved critical edge and texture information, CAM acted as an interpretable feature filter to highlight fault signatures, and the IRB backbone refined these features deeply. This coordinated multi-stage refinement, which was absent in ResCNN, MobileNet, and even Vision-Transformer, made the model particularly sensitive to discriminative fault patterns under varying operational conditions, ultimately leading to higher and more stable classification accuracy.

### 4.5. Transfer Experiment Research on SEU Dataset

To verify the effectiveness of the proposed method, the datasets set up in the previous sections were selected as the data sources. The CNN method combined with transfer learning (CNN + TL) [25], the fault diagnosis method based on Bidirectional Gate Recurrent Unit (BiGRU) [26], and the ResNet method combined with transfer learning (ResNet+TL) were selected as comparative experimental methods. To exclude randomness, each experiment was repeated 10 times. The optimizer of the experiment was set to Adam, the loss function was set to cross-entropy, the classifier was set to Softmax, the learning rate was set to 0.0001, the batch size was set to 32, and the number of iterations was set to 50.

For the SEU dataset, the following 4 transfer experiments were performed, A0-A2, A2-A0, B0-B2, and B2-B0, where A0-A2 means the A0 dataset was used as the source domain dataset and the A2 dataset was used as the target domain dataset for the transfer experiment. The accuracy of the 4 transfer experiments is shown in Table 11. It can be seen from the data in the table that the accuracy of the method proposed in this section was higher than that of other models in the 4 transfer tasks. The fault diagnosis accuracy of the ResNet+TL method was close to that of the method proposed in this section, but there was still a certain gap. The accuracy of the model proposed in this section was 100%, which means that all states could be accurately predicted, and the precision and recall of the model reached 100%, enabling high-precision recognition of all states. To prove the effectiveness of the model, the accuracy of the model without using transfer learning during training was compared with that of the proposed method.

**Figure 18:**
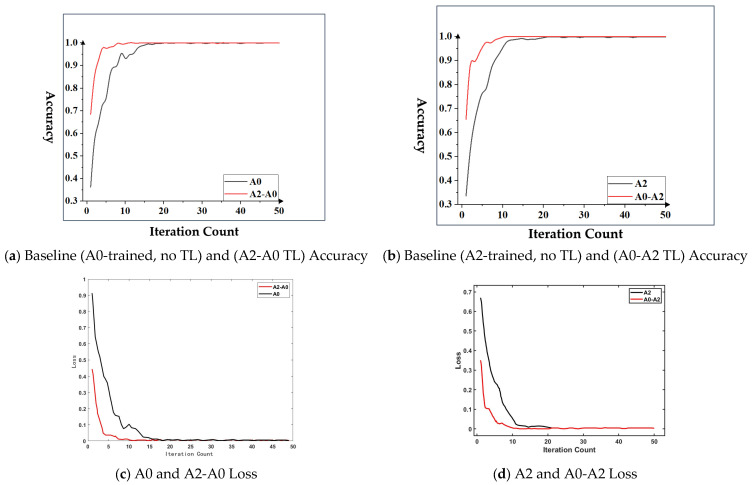
Comparison of training accuracy and loss for transfer tasks: (**a**) A0-trained baseline vs A2-A0 TL; (**b**) A2-trained baseline vs A0-A2 TL; (**c**) A2-A0 TL training/validation loss; (**d**) A0-A2 TL training/validation loss.

It can be seen from Figure 18a that, in the transfer task A2-A0, the proposed method achieved the highest accuracy at the 10th iteration, with a certain degree of fluctuation in the subsequent iterations, and the accuracy was 100%. Without using transfer learning, the model achieved the highest accuracy at the 20th iteration, which was 99.9%; in addition, in the transfer task, the initial accuracy of the model was 68.4%, while the initial accuracy of the model without using transfer learning was 36.2%. It can be seen from Figure 18b that the comparison between the transfer task A0-A2 and A2 was similar to that between A2-A0 and A0. Compared with the model without using transfer learning, the model using transfer learning had a much higher convergence speed, relatively less fluctuation after convergence, and a higher initial accuracy, which is more advantageous for fault diagnosis in emergency situations. From the above analysis, it can be seen that, compared with other selected fault diagnosis methods under the SEU dataset, the method proposed in this section has higher accuracy, faster convergence speed, and higher initial accuracy, making it more suitable for cross-working-condition fault diagnosis research.

It can be seen from Figure 18c,d that the training loss and validation loss of the transfer learning model show consistent downward trends throughout the training process. There is no obvious deviation between the two curves, indicating that the model does not suffer from overfitting. In contrast, the model without transfer learning exhibits slower loss convergence and larger fluctuations in the validation loss, which confirms that the proposed method’s rapid convergence and high accuracy stem from effective knowledge transfer, rather than data leakage.

**Figure 19:**
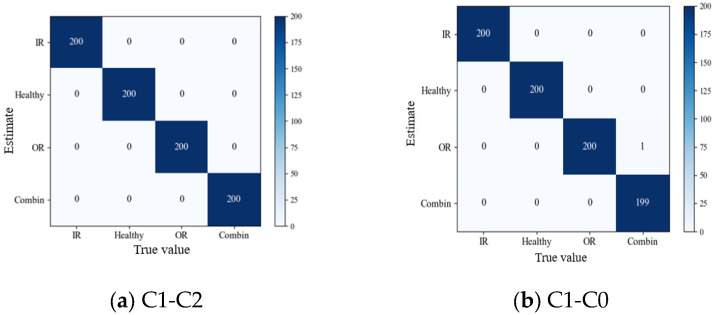
Confusion matrix of XJTU dataset migration experiment. (**a**) Confusion matrix of the transfer task C1-C2. (**b**) Confusion matrix of the transfer task C1-C0.

For the XJTU dataset, the following 2 transfer experiments were performed: C1-C0 and C1-C2. The accuracy of the 2 transfer experiments is shown in Table 12. It can be seen from the data in the Table that the accuracy of the method proposed in this section was higher than that of other models for the 2 transfer tasks. To further study the performance of the network proposed in this section, confusion matrices were drawn using the results of the 2 transfer tasks, as shown in Figure 19.

**Figure 20:**
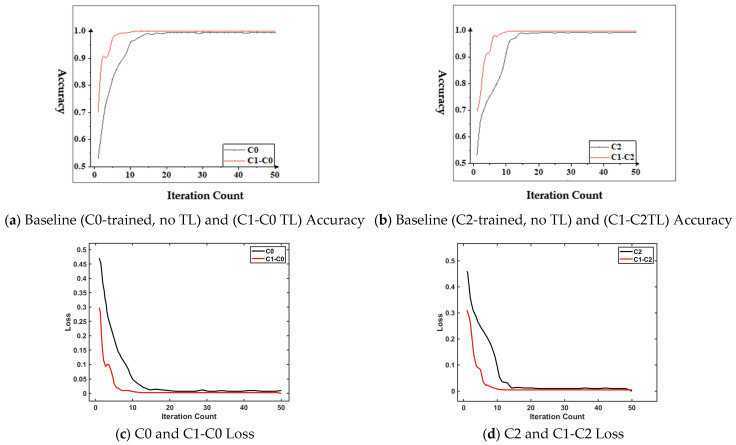
Comparison of the accuracy of the training processes. (**a**) Accuracy comparison between the model trained on dataset C0 and the transfer learning model (C1-C0). (**b**) Accuracy comparison between the model trained on dataset C2 and the transfer learning model (C1-C2). (**c**) Training and validation loss curves of the transfer learning model (C1-C0). (**d**) Training and validation loss curves of the transfer learning model (C1-C2).

It can be seen from Figure 19 (the confusion matrices of XJTU dataset transfer experiments) that the proposed method achieved accurate prediction of all states in the transfer task C1-C0 (Figure 19b). In the transfer task C1-C2 (Figure 19a), due to the existence of compound faults involving the inner ring, outer ring, cage, and rolling elements, the recognition performance of the proposed method for compound faults decreased slightly, with only 1 sample misclassified as an outer ring fault. To further verify the effectiveness of integrating transfer learning, the accuracy of the model trained without transfer learning was compared with that of the proposed method, and the comparison results are shown in Figure 20.

It can be seen from Figure 20a that in the transfer task C1-C0, the method proposed in this section achieved the highest accuracy at the 10th iteration, with a small amount of fluctuation in the subsequent iterations, and the stable accuracy was 100%. Without using transfer learning, the model achieved the highest accuracy at the 20th iteration, with a certain degree of fluctuation in the subsequent iterations, and the stable accuracy was 99.5%; in addition, in the transfer task, the initial accuracy of the model was 70.3%, while the initial accuracy of the model without using transfer learning was 53%. It can be seen from Figure 20b that the comparison between the transfer task C2 and C1-C2 was similar to that between C0 and C1-C0. Compared with the model without using transfer learning, the model using transfer learning had a much higher convergence speed, relatively less fluctuation after convergence, and a higher initial accuracy, which is more advantageous for fault diagnosis in emergency situations. From the above analysis, it can be seen that, compared with other selected fault diagnosis methods under the XJTU dataset, the method proposed in this section had higher accuracy, faster convergence speed, and higher initial accuracy, making it more suitable for cross-working-condition fault diagnosis research. As shown in Figure 20c,d, the training loss and validation loss of the transfer learning model (C1-C0 and C1-C2) converged stably to low levels, and the gap between the two curves remained small during the entire iteration process. This demonstrates that the model learned generalized fault features rather than memorizing signal segments, effectively responding to the concern of potential data leakage.

### 4.6. Transfer Experiment Research on QPZZ-II Dataset

**Figure 21:**
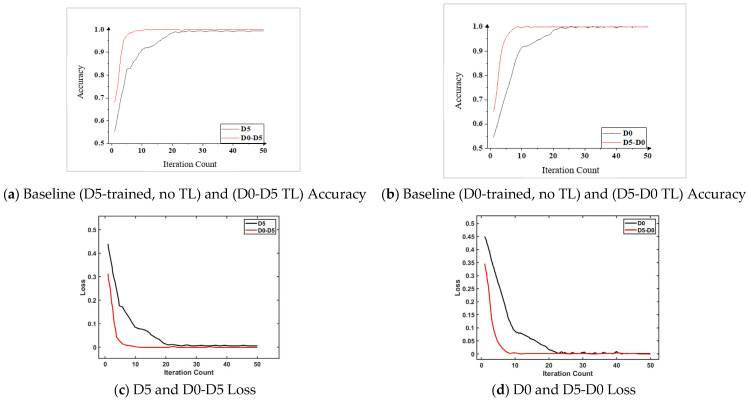
Comparison of the accuracy of the training process. (**a**) Accuracy comparison between the model trained on dataset D5 and the transfer learning model (D0-D5). (**b**) Accuracy comparison between the model trained on dataset D0 and the transfer learning model (D5-D0). (**c**) Training and validation loss curves of the transfer learning model (D0-D5). (**d**) Training and validation loss curves of the transfer learning model (D5-D0).

For the QPZZ-II dataset, the following 2 transfer experiments are performed: D0-D5 and D5-D0. The accuracy of the 2 transfer experiments is shown in Table 13. It can be seen from the data in the Table that the fault diagnosis accuracy of the proposed method in the 2 transfer tasks was 100%, which is higher than that of other models; that is, the proposed network can realize accurate prediction of all gear states. To prove the effectiveness of the model, the accuracy of the model without using transfer learning during training was compared with that of the proposed method. The comparison result is shown in Figure 21. It can be seen from Figure 21a that, in the transfer task D0-D5, the proposed method achieved the highest accuracy at the 10th iteration, with a certain degree of fluctuation in the subsequent iterations, and the accuracy was 100%. Without using transfer learning, the model achieved the highest accuracy at the 25th iteration, and the stable accuracy was 99.5%; in addition, in the transfer task, the initial accuracy of the model was 68.3%, while the initial accuracy of the model without using transfer learning was 55.4%. It can be seen from Figure 21b that the comparison between the transfer task D0-D5 and D5 was similar to that between D5-D0 and D0. Compared with the model without using transfer learning, the model using transfer learning had a much higher convergence speed, less fluctuation after convergence, and a higher initial accuracy, which is more advantageous for fault diagnosis in emergency situations.

From the above analysis, it can be seen that, compared with other selected methods under the QPZZ-II dataset, the proposed method had higher accuracy, faster convergence speed, and higher initial accuracy, making it more suitable for cross-working-condition bearing fault diagnosis research. From Figure 21c,d, it can be observed that the training loss and validation loss of the proposed method decreased synchronously and stabilized quickly. The validation loss does not show an upward trend, which fully verifies the model’s good generalization ability. The stable convergence of the loss curves further confirms the reliability of the 100% accuracy in transfer tasks.

## 5. Conclusions

Aiming at the limited ability of traditional neural networks to extract local fault features and their insufficient diagnostic accuracy under variable working conditions, this study proposes an intelligent diagnosis framework that integrates a Relative Position Matrix, a Downsampling Attention Module, and an Improved Residual Network. Transfer learning is further incorporated to address the challenge of knowledge transfer in small-sample scenarios. The main findings are as follows:

(1) Validation of Module Effectiveness: Ablation studies confirm that the combination of Haar wavelet downsampling and convolutional attention enables adaptive feature selection while preserving the key structural information of signals. The Improved Residual Module significantly enhances feature extraction efficiency and depth. The model demonstrates superior recognition performance for various fault states across multiple datasets.
(2) Outstanding Performance in Cross-Condition and Small-Sample Scenarios: The proposed framework was systematically tested under various combined conditions involving changing loads and speeds. It not only achieves high accuracy within a single working condition, but also maintains stable performance in cross-condition transfer tasks. Its accuracy is significantly higher than that of mainstream models used for comparison, such as ResNet, MobileNet, and Vision-Transformer. This indicates that the model learns generalizable fault representations, rather than memorizing signal patterns specific to a particular condition.
(3) Discussion on Experimental Design and Limitations: During data preparation, non-overlapping sliding windows were applied to signals from multiple independent trials to create samples, which were then pooled and randomly split. We acknowledge that, while this approach is a common benchmark for model comparison in the field, and all comparative models were trained and tested under this identical data splitting scheme, the chronological “block-wise” or trial-based “run-wise” splitting strategy suggested by the reviewers is theoretically more rigorous for evaluation. We will adopt this suggestion in subsequent work to further validate the temporal generalization capability of the method.

Future work will focus on the following aspects:

(1) Addressing More Complex Data Scenarios: Research will focus on diagnostic robustness under class imbalance and strong noise environments. Plans include introducing noise injection and signal-to-noise ratio tests, as well as validating the method’s effectiveness with real industrial data.
(2) Enhancing System Intelligence and Applicability: We will explore schemes for automated hyperparameter optimization and conduct research on open-set recognition. This aims to enable the model to detect unknown fault types, thereby better adapting to open and dynamic industrial environments.

## Figures and Tables

**Table 1:** Network architecture parameters of DAM-ImResNet.

Module Name	Input Size (H × W, C)	Operation Sequence (Per Building Block)	Repetitions	Output Size (H, W, C)
DAM	224 × 224, 3	7 × 7, 64	1	(56 × 56, 64)
Conv1	56 × 56, 64	$\left[\begin{array}{cc} 3\times 3 & ,64\\ 1\times 1 & ,256\\ 1\times 1 & ,64 \end{array}\right]$	×3	(56 × 56, 64)
Conv2	56 × 56, 64	$\left[\begin{array}{cc} 3\times 3 & ,128\\ 1\times 1 & ,512\\ 1\times 1 & ,128 \end{array}\right]$	×4	(28 × 28, 128)
Conv3	28 × 28, 128	$\left[\begin{array}{cc} 3\times 3 & ,256\\ 1\times 1 & ,1024\\ 1\times 1 & ,256 \end{array}\right]$	×6	(14 × 14, 256)
Conv4	14 × 14, 256	$\left[\begin{array}{cc} 3\times 3 & ,512\\ 1\times 1 & ,2048\\ 1\times 1 & ,512 \end{array}\right]$	×3	(7 × 7, 512)
Classification Module	7 × 7, 512	Global Average Pooling & Fully-Connected Layer	-	-

**Table 2:** Computational efficiency comparison.

Model	Params (M)	FLOPs (G)	Inference Time (ms)
ResNet50	25.6	4.1	12.3
DAM-IresNet50	18.7	3.2	9.8
Vision-Transformer	86.6	16.8	35.2

**Table 3:** Dataset D.

Dataset	Dataset Name	Fault Types	Working Condition
D	D0	Healthy, Gear Tooth Breakage, Root Crack, Surface Pitting, Gear Wear	No-load
	D5	Healthy, Gear Tooth Breakage, Root Crack, Surface Pitting, Gear Wear	Fixed load of 5 N·m

**Table 4:** Details of the SEU dataset.

Dataset Category	Dataset Name	Fault Types	Working Condition
A	A0	Healthy, Gear Tooth Breakage, Missing Gear Tooth, Root Crack, Surface Pitting	20 HZ–0 V
	A2	Healthy, Gear Tooth Breakage, Missing Gear Tooth, Root Crack, Surface Pitting	30 HZ–2 V
B	B0	Healthy, Inner Ring Wear, Outer Ring Wear, Rolling Element Fault, Combined Fault	20 HZ–0 V
	B2	Healthy, Inner Ring Wear, Outer Ring Wear, Rolling Element Fault, Combined Fault	30 HZ–2 V

Note: “20 HZ–0 V” indicates the working condition of motor frequency 20 Hz and no-load (load voltage 0 V); “30 HZ–2 V” indicates the working condition of motor frequency 30 Hz and load (load voltage 2 V).

**Table 5:** Details of the XJTU dataset.

Dataset Category	Dataset Name	Failure Locations	Working Condition
C	C0	Outer Ring, Combined Inner and Outer Rings, Healthy, Cage	2100 RPM, 12 KN
	C1	Outer Ring, Inner Ring, Healthy, Cage	2250 RPM, 11 KN
	C2	Healthy, Inner Ring, Outer Ring, Combined Inner and Outer Rings, Cage, Rolling Element	2400 RPM, 10 KN

**Table 6:** Classification results on the A0 dataset.

Fault Name	Number of Training Set Samples	Number of Validation Set Samples	Label
Gear Tooth Breakage	800	200	Chipped
Healthy	800	200	Health
Missing Gear Tooth	800	200	Miss
Root Crack	800	200	Root
Surface Pitting	800	200	Surface

**Table 7:** Different model settings.

Model Name	IRB	CBAM	HWD
ResNet50	No	No	No
IResNet50	Yes	No	No
CBAM-IresNet50	Yes	Yes	No
HWD-IresNet50	Yes	No	Yes
DAM-IresNet50	Yes	Yes	Yes

**Table 8:** Accuracy of ablation experiment under the same working conditions (%).

Working Condition	ResNet50	IResNet50	HWD-IResNet 50	CBAM-IresNet50	DAM-IresNet50
D0	96.4	97.5	98.4	99.2	100
D5	96	97.3	98	98.8	99.3

**Table 9:** Average accuracy of various models under variable working conditions (%).

Working Condition Change	ResCNN	MobileNet	Vision-Transformer	DAM-IResNet
A0-A2	91.5	95.4	96.7	98.9
A2-A0	90.3	93.8	97.5	98.6
B0-B2	89.7	94	95.4	97.5
B2-B0	91	92.5	96.8	98.1
C1-C0	91	92.1	96.4	97.2
C1-C2	90.7	90.6	95.4	96.6
D0-D5	92.5	95.7	98.2	99.3
D5-D0	92.1	95.2	98.4	99.0

**Table 10:** Evaluation indexes under D0-D5 variable working conditions.

Evaluation Index	Chipped	Healthy	Root	Surface	Wear
Precision	100	100	99.01	100	97.56
Recall	100	99	100	97.5	100

**Table 11:** Comparison of accuracy (%) of different models for SEU dataset transfer tasks.

Working Condition	CNN + TL	BiGRU	ResNet + TL	DAM-IResNet50 + TL
A0-A2	92	95	97.9	100
A2-A0	91.3	94.2	98.2	100
B0-B2	91.8	95.7	97.6	100
B2-B0	90.5	94.8	97.8	100

**Table 12:** Comparison of accuracy (%) of different models for XJTU dataset transfer tasks.

Working Condition	CNN + TL	BiGRU	ResNet + TL	DAM-IResNet50 + TL
C1-C0	86.5	90.5	97	100
C1-C2	85.7	91.2	96.8	99.75

**Table 13:** Comparison of accuracy (%) of different models for QPZZ-II dataset transfer tasks.

Working Condition	CNN + TL	BiGRU	ResNet + TL	DAM-IResNet50 + TL
D0-D5	90.5	93.5	98.2	100
D5-D0	89.2	94.5	97.8	100

## Data Availability

The data presented in this study are available on request from the corresponding author.
